# Examining Telemedicine Adoption After the COVID-19 Pandemic and Evaluating the Impact of a Virtual Hospital on Clinician Engagement: Longitudinal Survey Study

**DOI:** 10.2196/93911

**Published:** 2026-09-09

**Authors:** Galia Barkai, Shany Edelman-Yaron, Hadar Amir, Havi Inbar-Lankry, Rachel Sarafraz

**Affiliations:** 1 Sheba BEYOND Virtual Hospital Sheba Medical Center Ramat Gan Israel; 2 Gray Faculty of Medical & Health Sciences Tel Aviv University Tel-Aviv Israel; 3 Sheba Medical Center Ramat Gan, Tel Aviv Israel

**Keywords:** telemedicine, telehealth, health personnel attitudes, clinician engagement, clinician satisfaction, video consultations, implementation barriers, virtual hospital, change management

## Abstract

**Background:**

After the emergence of telemedicine as a major health care delivery mode during the COVID-19 pandemic, Sheba Medical Center (SMC) rapidly transitioned its outpatient clinics to telemedicine. A postimplementation satisfaction study revealed that while nearly 90% of patients reported a positive experience, only 38% of clinicians expressed high satisfaction. SMC soon established Sheba BEYOND, a dedicated virtual hospital. This initiative was accompanied by extensive efforts to improve the telemedicine experience. In 2025, a follow-up survey addressing clinician satisfaction and acceptance was conducted.

**Objective:**

This study aimed to explore whether clinician satisfaction improved and which barriers to clinician engagement persisted.

**Methods:**

An anonymous survey was distributed via a link to the staff of SMC’s outpatient clinics. The survey inquired about user experience with the telemedicine digital platform, integration in workflow, barriers to use, perceived benefit to patients, and overall satisfaction. Clinicians who never or rarely perform telemedicine visits were classified as nonusers; all others were classified as users. The questionnaire included 21 questions, 2 additional questions for nonusers, and 5 additional questions for users. Overall satisfaction was ranked on a 10-point Likert scale. Descriptive statistics were calculated using SPSS (version 29.02; IBM Corp) and Microsoft Excel. A chi-square test of independence was used for categorical variables. We used a 2-tailed *t* test, with a probability of <.05 considered statistically significant.

**Results:**

The survey was sent to 2735 clinicians, and 274 (10%) responded. Telemedicine users accounted for 52.6% (144/274). Among all respondents, 54.4% (149/274) were physicians, 30.3% (83/274) were paramedical staff, and 15.3% (42/274) were nurses. Clinical specialties across all respondents were internal medicine (36/274, 13%), psychiatry (34/274, 12%), pediatrics (36/274, 13%), oncology (31/274, 11%), and other specialties (137/274, 50%). Compared to the 2020 survey, a 41.9% increase was recorded in the number of users reporting a high level of satisfaction, from 37.7% (61/162) to 53.5% (77/144; *P*=.006). This increase was largely driven by a significant improvement in the oncology clinic, where users reporting a high level of satisfaction rose from 23.3% (10/43) to 63.6% (14/22; *P*=.01). In total, 64.4% (176/274) of respondents believed that telemedicine was advantageous for patient care and 66% (95/144) of users reported that telemedicine reduced their workload. Among the 103 video-visit users who answered the technical feedback questions, 62.1% (64/103) experienced problems in connecting to the patients’ network and 32% (33/103) experienced connectivity problems in SMC. In fact, 80.6% (83/103) reported that they had to switch to the telephone at least once. Most of the 98 nonusers (80/98, 81.6%) stated that they did not perform telemedicine because it was mostly clinically inappropriate.

**Conclusions:**

This study demonstrates the positive impact of institutional change management strategy in promoting clinician engagement with telemedicine. However, persistent technical barriers and varying engagement highlight opportunities for refinement.

## Introduction

The COVID-19 pandemic catalyzed an unprecedented transformation in health care delivery worldwide, with telemedicine emerging as a vital solution to maintain continuity of care during lockdowns and social distancing mandates [1]. At Sheba Medical Center (SMC), outpatient services rapidly transitioned to telemedicine in early 2020. A postimplementation survey revealed a definite contrast in satisfaction levels: while nearly 90% of patients reported a positive experience, only 38% of clinicians expressed high satisfaction, citing technical challenges, administrative burden, and difficulty integrating telemedicine into clinical workflows [2]. This discrepancy echoed broader global trends observed at the time: a high level of patient satisfaction [3-5] with usability challenges on the clinicians’ side [6]. Although these studies examined short-term satisfaction during the pandemic, there is limited evidence on whether sustained institutional change can lead to durable cultural shifts in clinician engagement with telemedicine.

Soon after the onset of the pandemic, the SMC established Sheba BEYOND, a dedicated virtual hospital that launched new telemedicine-based, hybrid care services, including home hospitalization, remote rehabilitation, and chronic patient management. In addition to ensuring continuity of care during the pandemic, Sheba BEYOND was established as part of a broader organizational strategy aimed at improving accessibility to specialist care, reducing geographic barriers, enhancing timeliness and continuity of ambulatory services, and supporting long-term digital transformation through hybrid models of care delivery. This initiative was accompanied by extensive efforts to facilitate clinician engagement and improve the telemedicine experience through the establishment of a video telemedicine helpdesk for technical support, frequent technical upgrades, interoperability improvements, administrative streamlining, and leadership training and incentives for telemedicine adoption.

Between 2020 and 2024, the number of yearly telemedicine visits at SMC more than doubled, increasing from approximately 60,000 in 2020 to >114,000 in 2024. Yearly patient experience surveys have continued to show high levels of patient satisfaction, with minor fluctuations over the years. In 2025, we conducted a follow-up survey addressing clinicians to assess whether the organizational efforts to facilitate telemedicine adoption translated into increased clinician satisfaction and acceptance over time.

Unlike prior studies focused primarily on short-term telemedicine implementation during the acute pandemic phase, this study examines long-term clinician engagement several years later within the context of an institutional virtual hospital framework and sustained organizational change efforts and explores whether the significant increase in telemedicine visits reflects a true cultural shift or if barriers to clinician engagement persist.

## Methods

### Overview

To assess the experience of telemedicine clinicians, an anonymous survey link was distributed through both text message and email to SMC’s outpatient clinic staff. The survey questions assessed users’ experiences interacting with the telemedicine digital platform, integration of telemedicine in clinician workflow, barriers to use, perceived benefit to patients, and overall satisfaction and thoughts on potential improvements to the service. Clinicians who reported that they never or rarely perform telemedicine visits were classified as nonusers; all others were classified as users. The questionnaire included 21 questions for all respondents, 2 additional questions for nonusers, and 5 additional questions for users. Three reminders, sent through both text message and email, spaced 1 week apart, were sent to the clinicians who had yet to respond to the survey.

### Eligibility

The study did not define formal patient-level inclusion or exclusion criteria, as the survey focused on clinician experience across existing outpatient telemedicine services routinely performed at the institution. Decisions regarding telemedicine appropriateness were made according to specialty-specific clinical judgment and institutional practice.

### Surveys

Overall satisfaction was ranked on a 10-point Likert scale [7]. Satisfaction ratings of 8 to 10 were considered as a high level of satisfaction, ratings of 4 to 7 as an intermediate level of satisfaction, and ratings of 1 to 3 as a low level of satisfaction. Responses to operational questions such as ease of use and adherence to appointment schedule were rated on a 5-point Likert scale, with scores of 4 to 5 considered high, 3 considered intermediate, and 1 to 2 considered low. For patient benefit, ratings of 4 to 5 were considered as a high level of support, 3 was considered as an intermediate level of support, and 1 to 2 were considered as a low level of support. Several survey items were adapted from the previously published institutional telemedicine survey conducted in 2020, while additional questions were developed by the investigators to assess workflow integration, operational barriers, and modality-specific experiences [2]. Comparison to the 2020 survey was made only when questions were similar in the 2025 survey. The questionnaires (translated from Hebrew) are presented in Multimedia Appendix 1.

### Statistical Analysis

Descriptive statistics were calculated with Microsoft Excel and SPSS software (version 29.02; IBM Corp). A chi-square test of independence was used for categorical variables. We used a 2-tailed *t* test, with a probability of <.05 considered statistically significant.

### Ethical Considerations

The study received approval from SMC’s institutional review board for retrospective analysis of prospectively collected data (2516-SMC-D). Participation in the survey was voluntary and anonymous. Completion of the questionnaire was considered implied informed consent. No identifiable personal information was collected.

## Results

The survey was distributed to 2735 clinicians and 274 responded, representing a 10% response rate. Of the 274 respondents, 61% (n=167) accessed the survey link via text message. Telemedicine users accounted for 52.5% (144/274) of the respondents. Clinical characteristics and mode of communication are presented in Table 1.

**Table 1:** Clinician characteristics and mode of communication (n=274).

			Clinicians, n (%)
**Clinician characteristics (n=274)**			
	**Clinical specialty**		
		Internal medicine	36 (13)
		Psychiatry	34 (12)
		Pediatrics	36 (13)
		Oncology	31 (11)
		Other specialties	137 (50)
	**Clinician type**		
		Physicians	149 (54.4)
		Paramedical staff	83 (30.3)
		Nurses	42 (15.3)
**Mode of communication (n=143^a^)**			
		Mostly video visits	69 (48.3)
		Both telephone and video	56 (39.2)
		Primarily telephone	14 (9.8)
		Alternative modes	4 (2.8)

^a^Notably, of the 143 clinicians who responded to the question regarding the mode of communication, none of the 19 psychiatrists indicated telephone visits as a significant feature of their telemedicine practice; 36.8% (7/19) of the psychiatrists engaged predominantly in video visits, while 63.2% (12/19) exclusively conducted video-based telemedicine visits.

### User Satisfaction: Comparison to 2020

**Figure 1:**
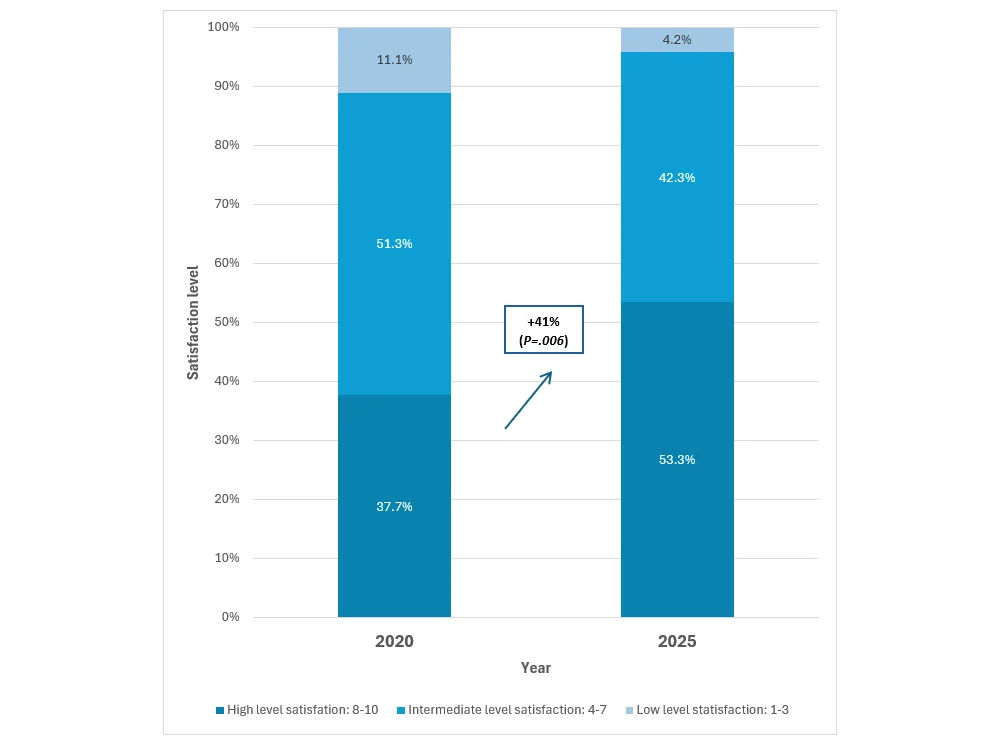
Comparison of overall user satisfaction in 2020 (n=162) and 2025 (n=144).

There was a 41.9% increase in users reporting a high level of satisfaction, from 37.7% (61/162) in 2020 to 53.5% (77/144) in 2025 (*P*=.006). Additionally, the proportion of respondents reporting a low level of satisfaction decreased from 11.1% in 2020 to 4.2% in 2025 (*P*=.02; Figure 1).

**Figure 2:**
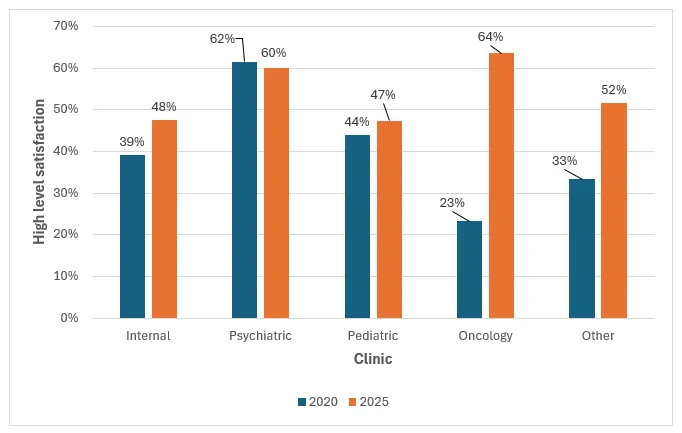
Comparison of numbers of users reporting a high level of satisfaction in different clinics in 2020 and 2025.

The observed overall increase in satisfaction was largely driven by a significant improvement among clinicians in the oncology clinic, where users reporting high satisfaction increased from 23.3% (10/43) in 2020 to 63.6% (14/22) in 2025 (*P*=.01). A high level of satisfaction in other clinics, including internal medicine, psychiatry, pediatrics, and other specialties, remained largely unchanged during the same period (Figure 2).

Changes in overall satisfaction levels across different sectors were predominantly nonsignificant. However, the high level of satisfaction among paramedical staff increased from 40.3% (29/72) to 59.3% (32/54; *P*=.03), while a low level of satisfaction among physicians decreased from 21.8% (19/87) to 9.5% (7/74; *P*=.03).

When queried about the perceived benefits of telemedicine for patients overall, 64.4% (176/274) of respondents expressed the belief that telemedicine was advantageous for patient care. No significant variation was observed across medical disciplines. Among telemedicine users, the proportion who considered telemedicine beneficial for their patients remained high and unchanged over time, at 87% (141/162) in 2020 and 86.6% (123/142) in 2025. In contrast, among nonusers, this proportion decreased from 68% (34/50) in 2020 to 41.9% (49/117) in 2025 (*P*<.001).

### Efficiency and Integration Into Workflow

The majority of users (95/144, 66%) reported that telemedicine reduced their workload and only 9% (13/144) felt their workload increased when performing telemedicine visits, with no significant differences observed across clinics. Nurses experienced the greatest benefit, with 90.5% (38/42) reporting reduced workload, compared to 63.8% (95/149) of physicians (*P*>.05) and 61.4% (51/83) of paramedical staff (*P*=.04).

Half of the users (73/144, 50.7%) felt that telemedicine visits were shorter and only 3.5% (5/143) of clinicians felt they were longer. Most internal medicine (16/21, 76.2%) and oncology (15/22, 68.2%) clinicians felt telemedicine visits were shorter, and most psychiatric (17/20, 85%) and pediatric (11/19, 57.9%) clinicians felt it took the same time as in-person visits.

Overall, 63.2% (91/144) of telemedicine users reported that patient appointments typically began on time. Punctuality rates were significantly higher in psychiatry (17/20, 85%) and pediatrics (15/19, 78.9%) compared to other specialties (37/62, 59.7%; *P*=.01). Clinicians who reported having delays (53/144, 36.8%) identified several factors contributing to the delays in telemedicine visits, including clinician-related delays (23/53, 43.4%), patient unavailability at the scheduled time (5/53, 9.4%), technical issues (12/53, 22.6%), and other miscellaneous reasons (13/53, 24.5%).

### Video Visits

Of the 143 users performing video visits, 51.7% (n=74) reported actively using SMC’s telemedicine platform (Datos Health), while 13.3% (n=19) indicated they had previously used it but discontinued and 35% (n=50) stated they had never used the platform. The most commonly used alternative video platforms were Zoom (Zoom Communications, Inc; 36/104, 34.6%) and WhatsApp (Meta Platforms, Inc; 18/104, 17.3%), with multiple responses permitted. Among the 103 video-visit users who answered the technical feedback questions, the Datos platform was considered very easy to use by 53.4% (55/103) of users, intermediately easy by 23.3% (24/103), and hard to use by 23.3% (24/103).

The proportion of clinicians performing video visits reporting technical or connectivity problems was high. Most clinicians (64/103, 62.1%) experienced problems in connecting to the patients’ network, 32% (33/103) experienced connectivity problems in SMC and only 7.8% (8/103) reported never having connectivity problems, with no significant differences among specialties. In fact, 80.6% (83/103) reported that they had to switch to telephone conversation at least once, and 23.3% (24/103) stated that it occurred at least half of the time. Only 19.4% (20/103) of users never had to switch to telephone.

A third (83/274, 30.3%) reported that they had contacted SMC’s helpdesk regarding technical difficulties with the video platform; 86.9% (238/274) stated that they were satisfied with the service they received from the helpdesk staff.

### Nonusers

In total, 20% (26/130) of nonuser respondents reported they had previously performed telemedicine and stopped. The proportion of clinicians who ceased to perform telemedicine visits was higher in psychiatry compared with other clinics although the absolute numbers were small (6/14, 42.9%). When asked for reasons for not performing telemedicine, of the 98 respondents who answered this question, 81.6% (n=80) stated that it was mostly clinically inappropriate, 23.4% (n=23) stated that they had experienced difficulties, 15.3% (n=15) thought it would interfere with their workflow, 9.2% (n=9) reported that they did not know how to perform telemedicine visits, and 8.2% (n=8) believed that patients would find it less convenient (multiple responses were allowed).

## Discussion

### Principal Findings

This study aimed to examine clinician engagement and satisfaction with telemedicine following institutional efforts to enhance uptake through technical improvements, administrative streamlining, and clinician support initiatives at SMC’s virtual hospital, Sheba BEYOND. The findings demonstrate that while significant progress has been made in improving clinician satisfaction, persistent obstacles continue to affect overall adoption.

The results demonstrate a significant improvement in clinician satisfaction with telemedicine services, with users reporting a high level of satisfaction increasing from 37.7% (61/162) in 2020 to 53.5% (77/144) in 2025 (*P*=.006), alongside a notable decrease in low levels of satisfaction.

The doubling of telemedicine visits between 2020 and 2024, along with a significant increase in clinician satisfaction levels, highlight a fundamental cultural shift in clinician attitudes toward telemedicine, reflecting the success of Sheba BEYOND’s robust infrastructure and multifaceted change management strategies. Similar interventions in other health care systems have successfully increased clinicians’ confidence in telemedicine platforms by addressing usability and logistical challenges and providing appropriate support [8]. Oncology clinicians showed the most substantial improvement in satisfaction (from 10/43, 23.3% to 14/22, 63.6%; *P*=.01), likely due to improvements to interoperability in the oncology clinic. It is possible that the increase is also due to exceptional receptiveness to telemedicine by oncology patients [9]. Satisfaction in psychiatry remained high; prior research has shown that psychiatrists believe most patients to be good candidates for telemedicine and that it is useful as a means to encourage patient engagement [10]. Satisfaction in pediatrics did not see meaningful change. Pediatric physicians may be suspicious of their ability to provide care and build rapport with patients when using telemedicine, a perception reported in other studies on pediatric telemedicine [11,12]. The greater improvement observed by paramedical staff may reflect the longitudinal nature of their clinical activities, which often include patient education and rehabilitation support that may integrate more naturally into telemedicine workflows. Physicians, in contrast, may remain more affected by limitations related to physical examination requirements and diagnostic complexity.

The sustained increase in telemedicine visits and improved satisfaction mirror findings from previous studies highlighting the importance of institutional frameworks in fostering a cultural shift toward telemedicine. For example, the study by Ramaswamy et al [4] reported similar trends in higher satisfaction and increased adoption when supported by extensive training, workflow compatibility, and leadership-driven initiatives. Sheba BEYOND’s multifaceted strategies align closely with global approaches to managing change in telemedicine adoption after the COVID-19 pandemic [8].

The results highlight a significant disparity in perceptions between telemedicine users and nonusers, with 87% (141/162) of users believing telemedicine benefits their patients compared to only 41.9% (49/117) of nonusers. Interestingly, confidence in telemedicine’s utility among nonusers decreased significantly, from 68% (34/50) in 2020 to 41.9% (49/117) in 2025 (*P*<.001). This decrease may not reflect a true change in perspective but rather be related to the fact that over time, clinicians who believe in the utility of telemedicine have gradually embraced it, leaving the “nonuser” group with those who continue to be skeptical about this mode of health care delivery. Nonusers repeatedly cited clinical inappropriateness (80/98, 81.6%), difficulties with workflow integration (15/98, 15.3%), and technical problems (23/98, 23.5%) as primary reasons for opting out of telemedicine services. The literature supports these concerns, noting that telemedicine may be less applicable to high-complexity or tactile specialties such as surgery or certain subspecialty consults [13].

These findings align with barriers highlighted in the study by McMaster et al [14], which identified difficulties in integrating telemedicine into traditional workflows and its appropriateness for certain specialties as key obstacles to clinician adoption. Two-thirds (95/144, 66%) of telemedicine users reported reduced workloads, and 50.7% (73/144) perceived telemedicine visits as shorter than in-person consultations. Internal medicine (16/21, 76.2%) and oncology (15/22, 68.2%) clinicians were particularly likely to report shorter visits, whereas psychiatry (17/20, 85%) and pediatrics (11/19, 57.9%) found visit durations comparable to in-person care. Such efficiency gains are consistent with studies showing that telemedicine facilitates streamlined care delivery [15].

However, adherence to scheduled appointment times remained a challenge, with only 63.2% (91/144) of telemedicine users reporting that visits typically began on time. Punctuality rates varied across specialties, with psychiatry (17/20, 85%) and pediatrics (15/19, 78.9%) performing better than others. Clinician-related delays (23/53, 43.4%) and technical problems (12/53, 22.6%) were identified as the main contributors to scheduling inefficiencies, emphasizing the need for ongoing optimization of workflows and technology.

A significant proportion of telemedicine users performing video visits reported connectivity issues, with 62.1% (64/103) experiencing problems with patients’ networks and 32% (33/103) encountering connectivity problems within SMC’s network. This constrained user experience led to 80.6% (83/103) of clinicians resorting to telephone consultations at least once, undermining the platform’s reliability. While SMC’s telemedicine platform was deemed very easy to use by 53.4% (55/103) of users, alternative platforms such as Zoom and WhatsApp enjoyed widespread use, albeit with varying consistency. SMC encourages the use of its institutional telemedicine platform but does not restrict clinicians from using alternative video communication platforms. The study design did not allow assessment of the reasons underlying clinicians’ preference for telephone versus video consultations. We speculate that some clinicians may have favored telephone visits due to their simplicity and reduced vulnerability to connectivity issues, while others may have preferred video visits because visual interaction was important for clinical assessment and patient communication.

The literature emphasizes the importance of stable and user-friendly telemedicine platforms in ensuring clinician satisfaction and adoption. A systematic review by Pogorzelska and Chlabicz [3] identified platform usability and seamless integration into clinical workflows as critical factors influencing telemedicine success during the pandemic. For institutions with large-scale telemedicine implementation, customized solutions that address specialty-specific needs, such as pediatric consultations or psychiatric evaluations, can significantly enhance adoption.

Institutional efforts to support telemedicine adoption must go beyond technology upgrades and training. Telemedicine adoption may be encouraged or hindered by specialty-specific concerns. To ensure wider engagement, there is a need to address clinician skepticism regarding the clinical appropriateness of telemedicine and to tailor solutions to their specialty needs and circumstances. SMC’s institutional efforts to expand telemedicine adoption, specifically in the nonuser group, continue to focus on specialty-specific workflow adaptation, training initiatives, and hybrid care model development, while recognizing that telemedicine may remain less suitable for certain clinical scenarios and specialties.

Additionally, addressing technical barriers is paramount. Streamlined software platforms, increased access to stable internet connections, and ongoing support are essential to improving reliability and user satisfaction [8]. Patient education and technological assistance can also mitigate connectivity issues, helping reduce clinician workload related to troubleshooting technical problems during visits.

This study has several limitations. Its 10% response rate may limit representativeness, as clinicians more actively engaged with telemedicine may have been more likely to respond. The study was conducted at a single tertiary medical center, which may limit generalizability. In addition, the study design did not allow correlation between clinician satisfaction and objective telemedicine use metrics at the individual level. Furthermore, patient experience was outside the scope of this clinician-focused survey. Future studies should aim to address these gaps with broader sampling and data regarding clinician uptake.

### Conclusions

This study demonstrates the positive impact of institutional change management strategy in promoting clinician engagement with telemedicine services. The doubling of telemedicine visits and significant improvements in clinician satisfaction reflect an adaptive cultural shift, driven by organizational commitment and infrastructure development. However, persistent technical barriers and varying specialty-specific engagement highlight opportunities for refinement. Addressing these challenges through targeted interventions is crucial to achieve sustained engagement and maximize telemedicine’s potential in outpatient care.

## Appendix

Multimedia Appendix 1 Survey questionnaire (translated to English).

## Abbreviations

SMC: Sheba Medical Center

## Data Availability

The datasets generated or analyzed during this study are available from the corresponding author on reasonable request.
