# Supplementary material for: Examining Telemedicine Adoption After the COVID-19 Pandemic and Evaluating the Impact of a Virtual Hospital on Clinician Engagement: Longitudinal Survey Study
Source: JMIR Hum Factors. 2026 Sep 9;13:e93911. doi: 10.2196/93911 (PMC13601868; doi:10.2196/93911)
Supplement: Supplementary file 1 [file humanfactors_v13i1e93911_app1.docx]

Survey on Treating Physician Experience Using Telemedicine in Outpatient Clinics

Via link sent by SMS / Email

As part of service improvement processes and the implementation of remote medicine, we would appreciate your response to a short questionnaire, from which we will learn and continue to improve.

Your opinion as a physician/therapist is important to us.

Survey link

The questionnaire is written in masculine form, but is intended for all genders.

(No option to refuse or skip questions)

(Question for all respondents)

1. **How many of your patient visits at the clinic do you usually conduct remotely? (Phone or video calls)**
1. I do not conduct remote visits
2. I rarely conduct remote visits
3. I conduct up to 1/4 of visits remotely
4. I conduct between 1/4 and 1/2 of visits remotely
5. More than 1/2 of visits are conducted remotely

(For respondents who answered 3-4-5 Question 1)

2. **Please rate your overall satisfaction with conducting a remote visit on a scale of 1–10, where 10 means excellent and 1 means very poor**

1 2 3 4 5 6 7 8 9 10

(For respondents who answered 3–4–5 in Question 1)

3. **To what extent does conducting a remote visit make it harder or easier for you to perform your work?**

1 – Makes it very difficult	5 – Makes it very easy

1 2 3 4 5

(For respondents who answered 1–2 in Question 1 – non-users)

4. **Have you conducted remote visits in the past and stopped?**
1. Yes
2. No
3. Other ____________

(For respondents who answered 1–2 in Question 1 – non-users)

5. **Why do you not conduct remote visits or conduct them infrequently? (More than one answer may be selected)**
1. I do not know how to conduct remote visits
2. It makes my work harder
3. It makes it harder for the patient
4. Technical difficulties / malfunctions / complexity
5. Usually not clinically appropriate
6. Slows down my work and delays appointments
7. Difficult to manage remote calls while simultaneously seeing patients physically
8. Other ____________

(For respondents who answered 3–4–5 in Question 1)

6. **Is the remote visit usually conducted at the scheduled time?**
1. Usually yes
2. Usually no
3. Not consistent

(For those who rated 2–3 above)

7. **What is the main reason appointments do not take place on time?**
1. Delays on my part
2. Patient delays / unavailable
3. Technical delays
4. Other ____________

(For respondents who answered 3–4–5 in Question 1)

8. **Compared to an in-person appointment, is a remote appointment:**
1. Shorter
2. Longer
3. Same duration

(For respondents who answered 3–4–5 in Question 1)

9. **How do you conduct remote visits?**
1. Phone only
2. Mainly phone, some video
3. Equal phone and video
4. Mainly video, some phone
5. Video only
6. Other ____________

(For all respondents)

10. **Have you used the DATOS system for remote care?**
1. Have not used DATOS
2. Used and stopped
3. Use as needed
4. Other ____________
11. **Is there another platform, besides DATOS, that you use for video calls?**

**(More than one answer may be selected)**

1. WhatsApp Video
2. Zoom
3. Other ______
4. Not relevant

(For DATOS users – Question 10 answers 2 and above)

12. **How easy and convenient is it to conduct video calls in DATOS?**

**1 – Very complicated	5 – Very easy**

1 2 3 4 5

13. **Have you ever started a DATOS video call and switched to a phone call?**
1. Never
2. Occasionally
3. Half the time
4. Most of the time
5. Almost always

(For DATOS users – Question 10 answers 2 and above)

14. **Did you or the patient experience any of the following issues?**

(More than one answer may be selected)

1. No issues
2. My network connection problem
3. Patient network connection problem
4. Could not hear me
5. Could not hear patient
6. Could not see me
7. Could not see patient
8. Other ____________

(New question – for all)

15. **Did you receive training regarding remote visits and DATOS usage?**
1. Yes
2. No

(New question – for all)

16. **Have you contacted Beyond technical support regarding DATOS?**
1. Yes
2. No

If yes:

17. **How satisfied were you with the technical support service?**

**1 – Not satisfied	5 – Very satisfied**

1 2 3 4 5

If rated 3 or below:

18. **What were you dissatisfied with?**

(More than one answer may be selected)

1. Not available
2. Not courteous
3. Problem not solved
4. Other ____________

(For all respondents)

19. **To what extent do you believe remote visits benefit patients?**

1 – No benefit	5 – Great benefit

(For all respondents)

20. **What improvements or additions would improve the remote visit process?**

_________________________________________________________________________

(For all respondents)

21. **Additional comments or suggestions:**

________________________________________________________________________________________________
